# Variation in clinical target volume delineation in postoperative radiotherapy for biliary tract cancer

**DOI:** 10.1371/journal.pone.0273395

**Published:** 2022-09-01

**Authors:** Taeryool Koo, Kwang-Ho Cheong, Kyubo Kim, Hae Jin Park, Younghee Park, Hyeon Kang Koh, Byoung Hyuck Kim, Eunji Kim, Kyung Su Kim, Jin Hwa Choi

**Affiliations:** 1 Department of Radiation Oncology, Hallym University College of Medicine, Anyang, Republic of Korea; 2 Department of Radiation Oncology, Ewha Womans University Mokdong Hospital, Ewha Womans University College of Medicine, Seoul, Republic of Korea; 3 Department of Radiation Oncology, Hanyang University College of Medicine, Seoul, Republic of Korea; 4 Department of Radiation Oncology, Soonchunhyang University Seoul Hospital, Soonchunhyang University College of Medicine, Seoul, Republic of Korea; 5 Department of Radiation Oncology, Konkuk University School of Medicine, Seoul, Republic of Korea; 6 Department of Radiation Oncology, Seoul Metropolitan Government Seoul National University Boramae Medical Center, Seoul, Republic of Korea; 7 Department of Radiation Oncology, Korea Institute of Radiological and Medical Sciences, Seoul, Republic of Korea; 8 Department of Radiation Oncology, Ewha Womans University Seoul Hospital, Ewha Womans University College of Medicine, Seoul, Republic of Korea; 9 Department of Radiology, Chung-Ang University College of Medicine, Seoul, Republic of Korea; IRCCS Giovanni Paolo II Cancer Hospital, ITALY

## Abstract

We aimed to evaluate the inter-clinician variability in the clinical target volume (CTV) for postoperative radiotherapy (PORT) for biliary tract cancer (BTC) including extrahepatic bile duct cancer (EBDC) and gallbladder cancer (GBC). Nine experienced radiation oncologists delineated PORT CTVs for distal EBDC (pT2N1), proximal EBDC (pT2bN1) and GBC (pT2bN1) patients. The expectation maximization algorithm for Simultaneous Truth and Performance Level Estimation (STAPLE) was used to quantify expert agreements. We generated volumes with a confidence level of 80% to compare the maximum distance to each CTV in six directions. The degree of agreement was moderate; overall kappa values were 0.573 for distal EBDC, 0.513 for proximal EBDC, and 0.511 for GBC. In the distal EBDC, a larger variation was noted in the right, post, and inferior direction. In the proximal EBDC, all borders except the right and left direction showed a larger variation. In the GBC, a larger variation was found in the anterior, posterior, and inferior direction. The posterior and inferior borders were the common area having discrepancies, associated with the insufficient coverage of the paraaortic node. A consensus guideline is needed to reduce inter-clinician variability in the CTVs and adequate coverage of regional lymph node area.

## Introduction

Biliary tract cancer (BTC) arises in the bile duct system being surrounded by numerous critical organs and major vessels. The anatomical location and spreading pattern make a complete resection a complex procedure. Jarnagin et al. [1] reported that only a half of patients who were considered to be resectable received a potentially curative resection. For advanced BTC patients who are not suitable for curative surgery, systemic treatments consisting of cisplatin plus gemcitabine are preferred [2].

Significant differences in survival are found according to the resection status. The 5-year survival rates are up to 50% with a complete resection, but decrease to as low as 0% with an incomplete resection or without resection [3–7]. To improve prognosis of advanced BTC, immunotherapeutic agents can be added to the first-line treatment [8]. Also, optimal second-line systemic treatments after progression of advanced BTC are under exploring, such as adding oxaliplatin or liposomal irinotecan to fluorouracil and folinic acid [9, 10].

Regarding the patterns of failure, locoregional failure (LRF) has been reported to occur as the first recurrence. In extrahepatic bile duct cancer (EBDC) patients undergoing curative resection without adjuvant RT, about 40% of patients experienced LRF [11–13]. Similarly, gallbladder cancer (GBC) patients have been reported that LRF occurred in 30–40% of patients as an initial failure, although the incidence of distant failure was relatively higher than EBDC patients [14, 15].

Considering the patterns of failure, it is a rational strategy to add radiotherapy (RT) to reduce LRF. Owing to the rareness of BTC, accounting for 3% of malignancies in the gastrointestinal (GI) system [16], there are no randomized controlled trials confirming the benefit of postoperative RT (PORT). Although the evidence of PORT is not clear, BTC patients with unfavorable risk factors were recommended to undergo PORT concomitantly with fluoropyrimidine-based chemotherapy [17].

However, clinical target volume (CTV) guidelines for PORT have not yet been established in BTC. There are a few reports proposing contouring guidelines for upper abdominal normal organs [18] or CTVs of definitive RT in patients with locally advanced BTC [19]. Physicians inevitably extrapolate from the CTV guideline for PORT of pancreatic head cancer [20]. Consequently, there can be variations in the CTVs among radiation oncologists. In the current study, we aimed to evaluate the inter-physician variability in the CTV contouring for resected BTC.

## Materials and methods

Medical records of patients who underwent curative resection followed by PORT between 2016 and 2021 for distal EBDC (dEBDC), proximal EBDC (pEBDC), and GBC were reviewed. We found thirteen dEBDC patients undergoing pylorus-preserving pancreaticoduodenectomy for dEBDC, twelve pEBDC patients undergoing hepatobiliary resection, and seven GBC patients undergoing extended cholecystectomy. Then we selected those patients pathologically diagnosed with LN-positive and resection margin-negative diseases. Patients with involved resection margin were excluded to minimize viability resulted from the consideration about microscopic tumor extension. The number of patients who met these criteria was six for dEBDC, three for pEBDC, and five for GBC. Finally three BTC patients representing three tumor sites were selected, who had well-localized primary tumor and better image quality in computed tomography (CT) scans.

Table 1 details three BTC patients who underwent curative resection followed by PORT. Patient 1 underwent pylorus-preserving pancreaticoduodenectomy for dEBDC. The primary tumor invaded the bile duct wall with a depth of 5-12mm (pT2), and lymph node (LN) metastases were confirmed in two out of 11 LNs (pN1). Patients 2 received extended hemihepatectomy for pEBDC. The primary tumor invaded adjacent hepatic parenchyma (pT2b), and one out of seven LNs was found to be involved (pN1). Patient 3 had laparoscopic extended cholecystectomy for GBC. The primary tumor invaded perimuscular connective tissue on the hepatic side without extension into the liver (pT2b), and metastases were confirmed in one out of two LNs (pN1). Pathologic TNM staging was based on the 8th edition of the American Joint Committee on Cancer [21].

**Table 1:** Patent characteristics.

	Case 1	Case 2	Case 3
Location	Distal common bile duct	Left hepatic duct	Body of gallbladder
T stage	Tumor invades the bile duct wall with a depth of 5-12mm (pT2)	Tumor invades adjacent hepatic parenchyma (pT2b)	Tumor invades perimuscular connective tissue on the hepatic side, with no extension into the liver (pT2b)
N stage	Metastasis in 2 out of 11 LNs (pN1) Pericholedochal and peripancreatic LNs	Metastasis in 1 out of 7 LNs (pN1) Posterior pancreaticoduodenal LN	Metastasis in 1 out of 2 LNs (pN1) Posterior pancreaticoduodenal LN
Adjacent organs	Pancreas invasion (+) Duodenal invasion (-) Portal vein invasion (-) Hepatic artery invasion (-)	Gallbladder invasion (-)	Portal vein invasion (-) Hepatic artery invasion (-)
Resection margin	Free from tumor	Free from tumor	Free from tumor

LN, lymph node

The institutional review board approved this study (approval number: EUMC 2021-03-016), and waived for an informed consent. Medical history, preoperative abdomino-pelvic CTs, and pathologic reports were provided to nine radiation oncologists from nine institutions. All the radiation oncologists trained in the same institution, and have careers in radiation oncology for four to fourteen years. The radiation oncologists were asked to delineate CTVs on the free-breathing CT scans for RT planning, including primary tumor bed and regional LNs. The nine clinicians did not share protocols for BTC contouring or discuss about CTV delineation during the study period. The information of clinicians who contoured CTVs were masked, then CTVs were collected and analyzed.

We implemented data analysis to statistically verify the consensus among the CTVs delineated by the nine clinicians for each case. First, we calculated volumes of each CTV for each patient and minimum, maximum, mean, standard deviation, intersection, and union volume of CTVs per case using the “computational environment radiotherapy research (CERR)” [22]. The recent version of CERR contains a consensus tool for more probabilistic and quantitative analysis such as apparent agreement, kappa corrected agreement, and “simultaneous truth and performance level estimation (STAPLE)” based probabilities as well as sensitivity, specificity, and overall kappa value.

If the ‘true contour’ is assumed to be within the CTVs delineated by the nine clinicians, it might be between the intersection and union volume. Then, for each voxel in a union volume, it is possible to estimate the proportion of how many clinicians included the voxel. We can define the agreed volume based on a confidence level (CL); CL = 0% becomes a union, and CL = 100% becomes an intersection volume, theoretically. This factor is determined as an “apparent agreement” in the CERR. However, coincidence can be involved in the evaluation of the apparent agreement. Therefore, generalized kappa needs to be applied to evaluate the consistency regarding voxels being included in the target by chance. This concept is expressed in the following way:

$$Kappa=\frac{\left(Apparent\_agreement-Chance\_agreement\right)}{\left(1-Chance\_agreement\right)}$$,

where Chance_agreement is the expected agreement by chance alone and is based on marginal totals [23]. When the apparent agreement is corrected using the kappa value, it generally has less agreement than the apparent agreement.

The overall kappa value is often used as a single metric evaluation factor of concordance in target volume consensus studies. According to the value, the overall kappa is classified as follows: 0 indicates no agreement, 0–0.2 indicates slight agreement, 0.21–0.4 indicates fair, 0.41–0.6 indicates moderate, 0.61–0.8 indicates substantial, and more than 0.81 indicates excellent agreement [23].

STAPLE is known as the expectation maximization algorithm, which can decide the ‘true contours’ by optimizing sensitivity and specificity parameters of all contours per case [24]. The user can define the desired ‘true contours’ by adjusting CL. In this study, we generated CTV to have a CL of 80% (CTV80), which was used as the benchmark.

In addition, we computed borders of nine CTV and CTV80 per case in six directions (left-right (LR), anterior-posterior (AP), and superior-inferior (SI)). Then we calculated the discrepancy between the CTV80 and each CTV in six directions. We used in-house software for the analysis written in MATLAB (MathWorks, Natick, MA).

Another standard method to verify consistency is a conformity index (CI). We adopted generalized CI (CI_gen_) proposed by Kouwenhoven et al. to evaluate CI regardless of the number of observers without bias [25]. CI_gen_ is conceptually expressed as:

$${CI}_{gen}\frac{\sum_{pairsi,j}\left|A_{i}\cap A_{j}\right|}{\sum_{pairsi,j}\left|A_{i}\cup A_{j}\right|}$$,

where *A*_*i*_ and *A*_*j*_ stand for *i’th* and *j’th* clinician’s contour. Generally, if it is less than 0.5, the correlation is estimated as low, and if it is 0.7 or more, it is considered suitable.

## Results

A total of 27 CTVs for dEBDC, pEBDC, and GBC delineated by nine clinicians were analyzed (Table 2). The mean and standard deviation of CTVs were 120.62 ± 40.98 cm^3^ for dEBDC, 152.05 ± 54.84 cm^3^ for pEBDC, and 131.94 ± 46.93 cm^3^ for GBC. The degree of agreement was moderate in all cases; overall kappa values were 0.573 for dEBDC, 0.513 for pEBDC, and 0.511 for GBC. The CI_gen_ values were less than 0.5, and all cases showed weak correlation.

**Table 2:** Summary of clinical target volume (CTV) statistics.

Parameters	distal EBDC	proximal EBDC	GBC
Volume minimum (cm^3^)	52.65	57.41	73.95
Volume maximum (cm^3^)	174.37	253.78	227.70
Volume mean (cm^3^)	120.62	152.05	131.94
SD (cm^3^)	40.98	54.84	46.93
Volume union (cm^3^)	252.00	414.01	344.94
Volume intersection (cm^3^)	36.39	21.36	19.84
Volume of STAPLE generated contour with confidence level of 80%	137.24	189.43	164.75
Overall kappa^a^	0.573	0.513	0.511
Mean sensitivity	0.705	0.627	0.607
SD of sensitivity	0.205	0.177	0.133
Mean specificity	0.962	0.979	0.981
SD of specificity	0.035	0.024	0.024
p-value	0	0	0
CI_gen_^b^	0.487	0.372	0.369

SD, standard deviation; CI_gen_. generalized conformity index.

^a^Overall kappa value of 0 indicates no agreement, 0–0.2 indicates slight agreement, 0.21–0.4 indicates fair, 0.41–0.6 indicates moderate, 0.61–0.8 indicates substantial, and more than 0.81 indicates excellent agreement.

^b^CIgen value of <0.5 is generally considered a weak correlation, while ≥0.7 is suitable correlation.

**Fig 1:**
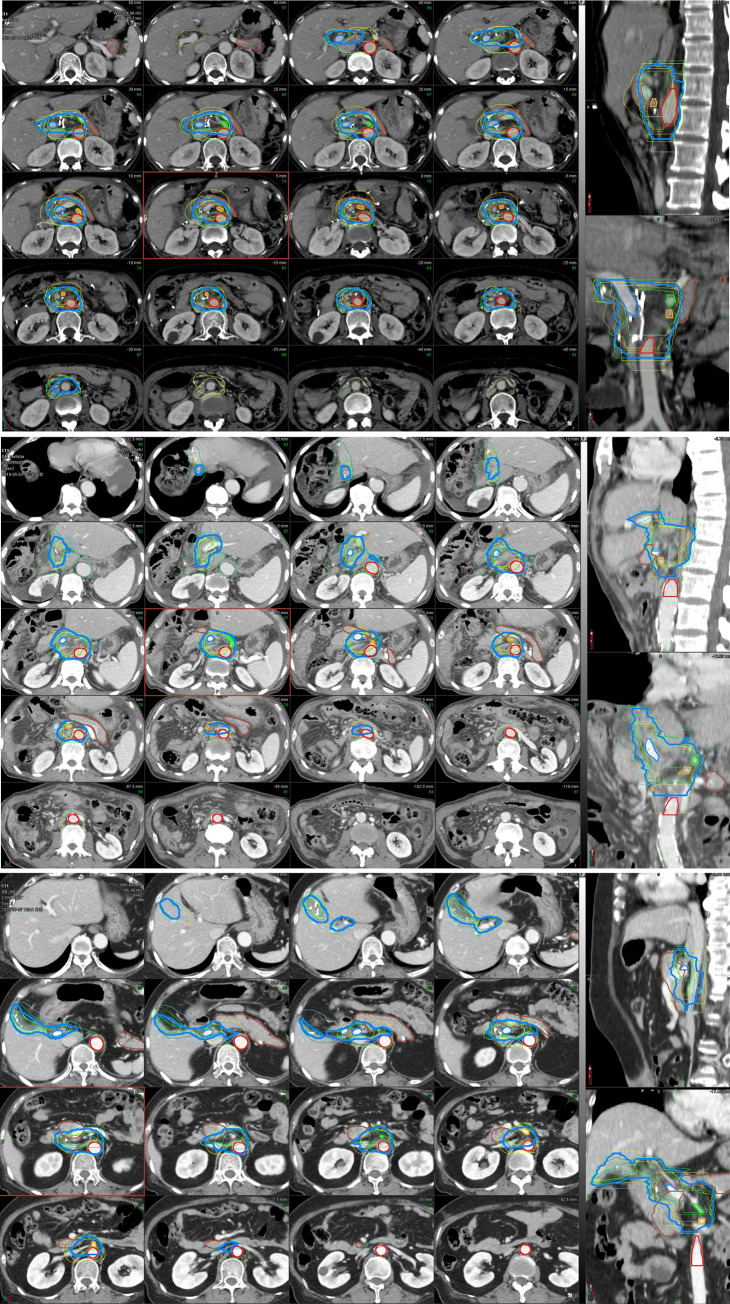
Clinical target volume (CTV) delineated by 9 radiation oncologists and CTV with confidence level of 80% (CTV80) generated by STAPLE. (A) distal extrahepatic bile duct cancer, (B) proximal extrahepatic bile duct cancer, and (C) gallbladder cancer. Magenta, CTV80; red, aorta; green, celiac artery; orange, superior mesenteric artery; blue, portal vein; and brown, pancreas.

**Fig 2:**
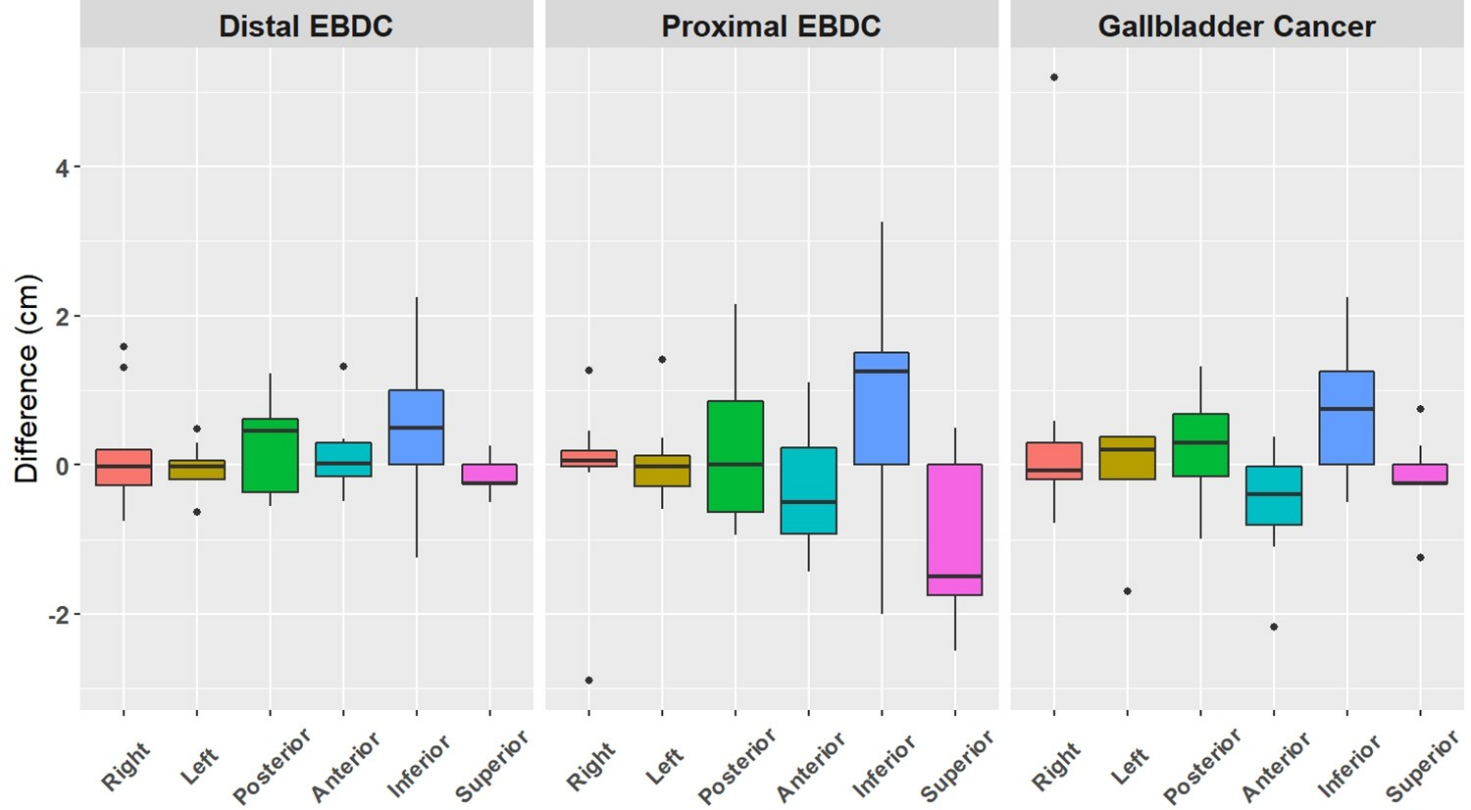
Plots of differences of borders in six directions between each clinical target volume (CTV) delineated by 9 radiation oncologists and CTV with confidence level of 80% generated by STAPLE.

CTV80 was generated and overlapped with the delineated CTVs in Fig 1. The differences in six directions borders between CTVs and CTV80 were plotted in Fig 2. In the dEBDC case, a relatively larger variation was noted in the right, post, and inferior directions. In the pEBDC case, all borders except the right and left directions showed larger variations. In the GBC case, a relatively larger variation was found in the anterior, posterior, and inferior borders. The posterior and inferior borders were the common areas showing larger discrepancies in all BTC cases.

## Discussion

The aim of PORT is to irradiate risky areas for LRF sufficiently while minimizing dose to the normal organs. With the introduction of intensity modulated RT (IMRT), more precise dose distribution can be achieved via accurate RT planning. Although the standardization of delineation guidelines is necessary to accompany the technical development, CTV guidelines for PORT of BTC have not yet been established. At this time, to the best of our knowledge, there are no studies reporting the variability among radiation oncologists in the CTV delineation for BTC patients undergoing curative resection. This is the first study presenting the large variation in the CTV delineation among experienced radiation oncologists.

CTV should encompass regional LNs as well as primary tumor beds. According to the surgical series analyzing LN involvement [26–29], the LN stations with a high risk of involvement for BTC are the hepatoduodenal ligament (HDL), celiac axis (CA), superior mesenteric artery (SMA), anterior pancreaticoduodenal nodes (aPDN), posterior pancreaticoduodenal nodes (pPDN), and paraaortic nodes (PAN). Notwithstanding the objective data from the pathological-surgical studies, several geographic misses could be found in the CTVs. Socha et al. [30] reviewed PORT studies for BTC, and classified which LNs were included in the CTVs. Compared with pathological-surgical data, the PAN was frequently missed in the CTVs for BTC. Other LNs at risk were also hardly included; SMA in dEBDC and GBC, aPDN in dEBDC, and pPDN in GBC.

In the present study, we could find similar geographic misses in the CTVs delineated by experienced clinicians (Table 3). First, the anterior border showed a relatively wider variation in pEBDC and GBC. The pPDN and SMA would be corresponding to the aforementioned area. More importantly, the posterior and inferior borders were confirmed to have prominent discrepancies in all cases. The LN station associated with this area is the PAN. Anatomically, the PAN encompasses LNs in the left latero-aortic and inter-aortico-venous regions as well as the anterior and posterior regions of the aorta [31]. For the delineation of the PAN, the Radiation Therapy Oncology Group recommends to use an asymmetric expansion from the contour of the aorta. The upper and lower limits are from the most cephalad to the CA to the bottom of the L2 vertebral body, although defined for pancreatic cancer [20]. In our study, however, participant experienced radiation oncologists frequently omitted the LNs in the posterior to the aorta or the inter-aortico-venous regions. The inferior margin of the PAN showed inconsistency, either (Fig 1).

**Table 3:** Regional lymph nodes station and borders showing a relatively wider variation.

Lymph node stations	Borders	Tumor location
Paraaortic	Posterior & inferior	All
Hepatoduodenal ligament	Right	Distal extrahepatic duct
Anterior pancreaticoduodenal	Anterior	Distal extrahepatic duct
Posterior pancreaticoduodenal	Anterior	Proximal extrahepatic duct & gallbladder
Superior mesenteric artery	Anterior	Gallbladder

However, PAN involvement is classified as distant metastasis (M1) in the TNM staging system for dEBDC and GBC [21, 32], and treatment outcomes are poorer in patients with PAN metastasis, comparing with other regional LN involvement. Furthermore, the surgical resection was generally accepted as a contraindication in BTC patients with PAN metastasis, as it is the last station of lymphatic pathways drained from the biliary system [33, 34]. In the meanwhile, several surgical series have been reported that extended lymphadenectomy could be helpful to improve prognosis in BTC patients with PAN metastasis [26, 35, 36]. Given the PAN is one of the most risky areas with 7–25% of BTC patients having the PAN involvement [30] further studies are needed to establish the optimal management for these patients. In addition, CTVs based on the risk of involvement of PAN in BTC patients according to the tumor locations and stages needs to be suggested, if PORT is given.

Although a consensus guideline for CTV delineation is an important key to reduce variability in CTVs, the marked anatomical variation after curative resection is the frustration to visualize as an atlas. Instead, we can adopt the recommendations for PORT for pancreatic cancer [20] or definitive RT for BTC [19]. These atlases recommend adding margins of 1.0–3.0 cm from the major vessels, such as the aorta, portal vein, CA, and SMA. After the expansion, the primary tumor bed considering individual disease extent will be added in the CTVs. Similarly, studies analyzing the patterns of failure visualize LRF sites by mapping around the reference vessels [11, 30, 37, 38]. Therefore, the CTV delineation based on the expansion from the key vessels would be helpful to decrease inter/intra-clinician variability.

Adopting artificial intelligence (AI) can be a way to the future of PORT, besides referring key vessels to delineate CTVs. In recent years, AI is actively introduced into every steps of RT from deformable image registration to treatment planning and quality assurance [39]. Clinical feasibility of automatic delineation of CTVs has been evaluated in the setting of PORT [40, 41]. The assistance of AI is expected to reduce inter-clinician variability and contouring time for PORT [42]. In the future, we may leap the inevitable hurdle to study BTC, rareness, with the development of AI based on the collection and analysis of big data [43].

Limitations of the present study are that we focused on the CTV delineation in this study, while other practical issues were not included, such as the PTV margin, RT technique, or dose prescription. These issues might be clues to investigate the inter-clinician variability. More importantly, CTV was not separately delineated as CTV for the primary tumor bed and CTV for regional LNs. The observed variations reflected discrepancies in the CTV for the primary tumor bed as well as for regional LNs. However, the present study is meaningful as it discusses the wide inter-clinician variability in the CTVs for BTC.

## Conclusions

Experienced radiation oncologists showed a moderate agreement in the delineation of CTVs for BTC. Among the six directions, the prominent variation was found in the posterior and inferior borders, associated with the PAN. The consensus guideline is needed to reduce inter-clinician variability in real-world practices.

## Supporting information

S1 File (XLSX)
